# Immobilization and Biochemical Properties of the Enantioselective Recombinant NStcI Esterase of *Aspergillus nidulans*

**DOI:** 10.1155/2013/928913

**Published:** 2013-05-27

**Authors:** Carolina Peña-Montes, María Elena Mondragón-Tintor, José Augusto Castro-Rodríguez, Ismael Bustos-Jaimes, Arturo Navarro-Ocaña, Amelia Farrés

**Affiliations:** ^1^Department of Food Science and Biotechnology, Faculty of Chemistry, National Autonomous University of Mexico (UNAM), 04510 Mexico City, DF, Mexico; ^2^Department of Biochemistry, Faculty of Medicine, UNAM, 04510 Mexico City, DF, Mexico

## Abstract

The recombinant NStcI *A. nidulans *esterase was adsorbed on Accurel MP1000, where protein yield and immobilization efficiency were 42.48% and 81.94%, respectively. Storage stability test at 4°C and RT showed 100% of residual activity after 40 days at both temperatures. The biocatalyst retains more than 70% of its initial activity after 3 cycles of repeated use. Biochemical properties of this new biocatalyst were obtained. Maximum activity was achieved at pH 11 and 30°C, while the best stability was observed with the pH between 9 and 11 at 40°C. NStcI thermostability was increased after immobilization, as it retained 47.5% of its initial activity after 1 h at 60°C, while the free enzyme under the same conditions displayed no activity. NStcI preserved 70% of its initial activity in 100% hexane after 72 h. Enzymatic kinetic resolution of (*R,S*)-1-phenylethanol was chosen as model reaction, using vinyl acetate as acyl donor. After optimization of reaction parameters, the highest possible conversion (42%) was reached at 37°C, *a*_w_ of 0.07, and 120 h of bioconversion in hexane with an enantiomeric excess of 71.7%. NStcI has selectivity for (*R*)-enantiomer. The obtained *E* value (31.3) is in the range considered useful to resolve enantiomeric mixtures.

## 1. Introduction

Carboxylic ester hydrolases (EC 3.1.1.x) (CEH) are a diverse group of hydrolases which split carboxylic acid esters in different types of molecules. Lipases (E.C. 3.1.1.3) and esterases (E.C. 3.1.1.1) are the main groups of natural biocatalysts that promote the ester bond cleavage and formation [1]. The most important feature that distinguishes lipases and esterases is the substrate specificity. Lipases preferentially hydrolyze water-insoluble esters such as triglycerides composed of long-chain fatty acids while esterases prefer short-chain acid triglycerides. Another distinction is based on protein structure, most lipases possess a hydrophobic domain (lid) covering the active site, a feature that is absent in esterases [2]. Moreover, esterases obey classical Michaelis-Menten kinetics, whereas lipases need a minimum substrate concentration before high activity is observed [3]. In this direction, esterases may offer advantages as catalysts over lipases in the absence of an interface. All aspects described above demonstrate how fundamentally significant esterases are for biotechnological processes and why the search for new biocatalysts has become very important.

Enzyme immobilization ensures recycling of the biocatalyst, allows easy product separation, and may improve performance of the enzyme [4]. The kinetic behavior of free enzymes is different from the one shown by immobilized forms due to conformational changes, steric hindrance, substrate and products partition, and microenvironmental and diffusion effects. Among commonly used immobilization methods, adsorption is the most used one because of its simplicity [5]. Adsorption of a protein onto a solid surface is a widely used method for enzyme immobilization. Different hydrolases have been immobilized on porous polypropylene Accurel [6–9].

Chiral intermediates and fine chemicals are in high demand both from pharmaceutical and agrochemical industries for the preparation of bulk drug compounds and agricultural products [10]. Chirality is a key factor in the safety and efficacy of many drug products and thus the production of single enantiomers of drug intermediates has become increasingly important in the pharmaceuticals industry [11]. The growth in single-enantiomer pharmaceuticals has produced several blockbuster compounds [2]. Chiral phenylethanol derivatives are important chiral building blocks for pharmaceuticals, agrochemicals, and natural products [12]. Additionally, low molecular weight esters derivatives from phenylethanol are responsible of fruity aroma and taste in food products [13].

On the other hand, there has been an increasing awareness of the enormous potential of microorganisms and enzymes derived therefrom for the transformation of synthetic chemicals with high chemo-, regio-, and enantio-selectivities [11]. CEH, especially lipases and esterases, play a crucial role in industrial esters synthesis. A considerable number of microbial carboxylesterases is known which have been overexpressed in suitable hosts; however, reports of esterases that have been used for synthesis of optically pure compounds are scarce. The major reasons for this are their limited commercial availability and their frequently observed moderate enantioselectivity [14, 15]. Esterases can be also employed in reactions where chemo- or regioselectivity is of interest instead of enantioselectivity [16–18]. Biocatalytic processes have many advantages over chemical ones, such as the chemio-, regio-, and enantioselective synthesis, which can be performed with low energy cost and enable a higher efficiency, especially in chiral compounds production [2]. Enantioselectivity is important in nature, as biological activities are not always performed by both enantiomers, like flavor compounds, which do not occur as a racemate but as one preferred enantiomer, resulting in a very different sensory profile depending on their enantioform. This is the key interest to produce specific products [19]. Over the last ten to fifteen years, drug chirality, particularly the use of single enantiomers versus racemic mixtures, has become an area of considerable interest. Of the new drugs introduced in 2000, 76% were single enantiomers compared with 21% in 1991 [20, 21]. Among the numerous synthetic methods, the enzyme-catalysed kinetic resolution of racemic alcohols through transesterification is an attractive route. Transesterification reactions catalysed by enzymes have been extensively studied in conventional non-aqueous medium [22–24]. Solvents define a major part of the environmental performance of processes in the chemical industry and have also an impact on cost, safety, and health issues [22, 25]. The use of organic solvents is especially advantageous to transform substrates that are unstable or poorly soluble in water. By varying the organic solvent, it is possible to control the substrate specificity as well as the regio- and enantioselectivity of a given enzyme. Water activity is another important factor; many side reactions that are water dependent can be prevented at low *a*_w_, including the denaturation of enzymes which, in organic media, can show a higher thermal stability. Synthesis of esters by CEH can be favored over hydrolysis in the absence of water [26, 27].

*Aspergillus nidulans* produces the esterase NStcI, which is involved in the biosynthesis of sterigmatocystin, a precursor of aflatoxins [28]. The nature of the reaction catalyzed by this enzyme, that is, deacetylation, suggests that NStcI may have the biocatalytic potential to perform reactions with high enantio- or regioselectivity [29]. Previously, we have cloned and expressed in *Pichia pastoris* the complete *nstcI *gene as well as a truncated version of the gene (*stcI*) reported by Brown et al. [28]. Both recombinant variants were tested for their ability to hydrolyze *p-*nitrophenyl esters and we observed differences in the abilities of these two enzymes to hydrolyze them. Different deacetylation reactions of bioactive compounds were also assayed and the results indicated differences in chemoselectivity for deacetylation of phenols, kojic acid, and flavonoid esters. We also observed differences in regioselectivity for the deacetylation of kojic acid and flavonoids. This behavior suggested that N-terminus of this enzyme plays an important role in its catalytic properties [29]. 

In the present work, we immobilized the recombinant NStcI protein on Accurel MP1000 and its biochemical properties and enantioselectivity were evaluated. The resolution of a racemic (*R,S*)-1-phenylethanol and the direct transesterification of (*R,S*)-1-phenylethanol with vinyl acetate were tested under different reaction conditions.

## 2. Materials and Methods

### 2.1. Expression of NStcI Esterase

*Pichia pastoris* growth was performed according to the techniques described in the manual of EasySelect Pichia Expression Kit (version G) (Invitrogen, Carlsbad, CA, USA). The expression of NStcI esterase in *P. pastoris* is described briefly as follows: recombinant clones were cultured in 25 mL of buffered glycerol-complex medium (BMGY) (1% yeast extract, 2% peptone, 100 mM potassium phosphate, pH 6.0, 1.34% YNB (yeast nitrogen base with ammonium sulfate and without amino acids), 4 × 10%–5% biotin, 1% glycerol) in a rotary shaker at 28°C and 250 rpm in 250 mL glass flasks. When cultures reached an OD_600_ = 2–6, the cells were centrifuged (3,000 ×g, 15 minutes). Esterase was induced by cell resuspension to an OD_600_ of 1 in 50 mL buffered methanol-complex medium (BMMY) which has the same composition as BMGY except for the use of 0.5% methanol instead of 1% glycerol. Cultures were again grown on a rotary shaker at 28°C, 250 rpm in 250 mL glass flasks during 96 h. Induction of esterase expression was achieved by daily addition of 5% methanol to a final theoretical concentration of 0.5%. After induction, *Pichia *cells were removed by centrifugation (3,000 ×g, 15 minutes), and supernatant was collected. Desalting, concentration (40-fold) and buffer exchange to phosphate buffer 50 mM and pH 7.5 of supernatant were done by ultrafiltration (Amicon, Beverly, MA, USA) through a membrane having 10-kDa NMWL (Millipore, USA). The concentrated solution was used to verify enzyme presence and its esterase activity. Esterase activity in the crude extract was detected by spectrophotometry at 410 nm, using *p*-NPA as substrate as described below. Protein analysis was done with a SDS-PAGE at 12% and the confirmation of its in situ esterase activity was carried out by zymography as described by Peña-Montes et al. [29].

### 2.2. Immobilization of NStcI Esterase

The recombinant enzyme was adsorbed on the microporous polypropylene support (Accurel MP1000) previously treated with ethanol 100% for 30 min, then with 50% ethanol for additional 30 min and washed with deionized water, which was removed by vacuum filtration; finally the biocatalyst was dried in a stove at 37°C for 2 h. Two strategies were evaluated: (a) 2 mg of protein of the enzymatic extract was added per gram of Accurel MP1000, and the mixture was incubated at 4°C with a shaking speed of 200 rpm on an orbital shaker for 48 h and (b) 1 mg of protein was added per gram of Accurel MP1000, and the mixture was incubated at 4°C with same agitation conditions during 0.25, 0.75, 1.5, 4, and 24 h. After that, the immobilized enzyme was separated by vacuum filtration on a Büchner funnel through filter paper (grade no. 1, Whatman), washed with 6 mL phosphate buffer (50 mM, pH 7.5) and the biocatalyst was dried in desiccator at 4°C for 2 h. The immobilization process was evaluated by taking aliquots from the initial and final solutions to quantify the protein content and enzyme activity as described below.

### 2.3. Protein Assay

Protein concentration was determined by the Bradford method [30] with a commercial protein assay kit (Bio-Rad Laboratories, Richmond, CA, USA), according to the manufacturer's instructions. Bovine serum albumin was used as the protein standard to construct the calibration curve. All results are presented as the mean of three assays.

### 2.4. Determination of Carboxylesterase Activity

Enzyme activity of crude extracts was quantified with *p-*NPA as described by Ejima et al. [31]. Esterase, 50 *μ*L, was added to 850 *μ*L of reaction buffer containing 50 mM potassium phosphate, pH 7.2, and 0.1% sodium deoxycholate. Finally, 100 *μ*L of 20 mM substrate (*p-*NPA) in methanol were added. Reaction was evaluated during 15 minutes. Each enzyme assay was performed in triplicate. The reaction was carried out at 25°C and 1 U of enzyme activity was defined as the quantity of enzyme that releases 1 *μ*mol of *p*-nitrophenol per min at 25°C. Absorbance was continuously measured at 410 nm. The reported molar extinction coefficient for *p-*NP under these conditions was 13,400 cm^−1^ M^−1^.

The biochemical characterization assays were done with the following protocol. Activity against *p*-nitrophenyl acetate (*p*-NPA) was scaled up in microtiter plate formats with a final reaction volume of 200 *µ*L. In the case of immobilized esterase (IE), IE (10 mg) was submerged in 900 *µ*L of 50 mM phosphate buffer pH 7.2 and 100 *µ*L of stock 1 mM de *p*-NPA in ethanol. The reaction was incubated under stirring with a shaking speed of 400 rpm on a thermomixer (Eppendorf, Hamburg, Germany) at RT. The reaction was stopped after 10 min by chilling on ice. An aliquot of 200 *µ*L was dispensed into 96-well microtiter plate and absorbance was measured at 410 nm. Solid material was removed by pipetting 200 *μ*L of supernatant taking care of not introducing solid particles on tip. All results are presented as the mean of three assays. In the case of free enzyme in crude extracts, first 170 *μ*L of phosphate buffer 50 mM pH 7.2 were dispensed in each well, then 20 *μ*L of stock 1 mM of *p*-NPA in ethanol were added, and finally 10 *μ*L of crude extracts. In order to investigate autohydrolysis of substrate, the enzyme was replaced by buffer. Each enzyme assay was performed in triplicate, and kinetic reaction was followed by measuring absorbance at 410 nm each minute during 10 min at RT. One unit of activity was defined as the amount of enzyme required to convert 1 *μ*mol of *p*-NPA to *p*-NP per minute under the specified conditions.

A calibration curve of optical density against *p-*NP concentration was used to estimate the formation of *p-*NP. The standard curve was prepared in ethanol with *p-*NP concentrations ranging from 25 to 200 *μ*mol and a molar extinction coefficient of 4,900 cm^−1^ M^−1^ was obtained. Absorbance was measured at 410 nm. A programmed protocol in the software Gen5 1.10 provided with the Epoch spectrophotometer (BioTeK, USA) was used.

### 2.5. Biochemical Characterization

#### 2.5.1. Effects of Temperature and Thermal Stability

To determine the optimum temperature for assaying esterase activity, the enzymatic reactions were incubated at temperatures ranging from 30 to 70°C. Esterase activity was evaluated as described above. To assess hydrolysis of substrate because of reaction media, the enzyme was replaced by buffer. All results are presented as the mean of three assays.

In order to determine thermal stability, 10 mg of support containing immobilized enzyme in 1 mL of phosphate buffer 50 mM pH 7.2 were incubated for 15, 30, and 60 min at various temperatures ranging from 30 to 70°C. After incubation, the reaction tubes were cooled to RT, and an aliquot was taken for evaluation of the residual activities, which were determined with the esterase assay described above. The percentage of residual activity was calculated by comparing the activity to that of the untreated control enzyme. These tests were all performed in triplicate. 

#### 2.5.2. Effects of pH and pH Stability

The effect of pH on the activity of the immobilized esterase was investigated using the standard esterase activity assay described before at pH values ranging from 5.0 to 10.0. The buffers used were 50 mM sodium acetate for the pH 5.0, 50 mM sodium phosphate for pH 6.0 to 7.0, 50 mM Tris-HCl for pH 8.0 to 9.0, and 50 mM CAPS for pH 10. A control without enzyme was also evaluated at each pH to evaluate substrate autohydrolysis caused by reaction medium. The pH stability was evaluated by incubating 10 mg of support containing immobilized esterase during 1 and 3 h at RT in 1 mL of the buffers described above at pH values ranging from 5.0 to 11.0. After incubation, the reaction tubes were cooled to RT, an aliquot was taken for evaluation of the residual activities as described above. The percentage of residual activity was calculated by comparing the activity to that of the untreated control enzyme. All results are presented as the mean of three assays.

#### 2.5.3. Substrate Specificity

Substrate specificity of immobilized esterase was investigated by replacing the *p*-NPA of the reaction described above with the following substrates: *p*-nitrophenyl butyrate (*p*-NPB), *p*-nitrophenyl decanoate (*p*-NPD), *p*-nitrophenyl laurate (*p*-NPL), *p*-nitrophenyl myristate (*p*-NPM), *p*-nitrophenyl palmitate (*p*-NPP), and *p*-nitrophenyl stearate (*p*-NPE). The experiments were conducted in 0.05 M Tris-HCl buffer at pH 9 at RT. These tests were carried out in triplicate.

#### 2.5.4. Effect of Solvent on Enzyme Stability

 In these experiments, solvents with different log *P* values were chosen. To evaluate enzyme stability in solvents, samples of 10 mg of immobilized esterase or 5 mg of lyophilized free enzyme were incubated for 72 h in 1 mL of 100% of hexane, diisopropyl ether, *t*-butanol or acetone, at 25, 37, and 50°C. The solvent was removed and then reaction mixture (1 mL) containing 2 mM of substrate prepared as described above was added to the biocatalyst to quantify the residual hydrolysis activity with *p-*NPA, as indicated in Section 2.4.

### 2.6. Operational and Storage Stability of Immobilized Carboxylesterase

The biocatalyst reuse was evaluated up to 7 times, with repeated washing with phosphate buffer 50 mM, pH 7.2 after each esterase activity assay. The reaction mixture (1 mL) containing 2 mM of substrate was prepared as described in a previous section and was added to 10 mg of immobilized enzyme. The residual activity was quantified according to the method described in Section 2.4. All results are presented as the mean of three assays.

The storage stability of the immobilized biocatalysts was tested over a period of 40 days at room temperature and 4°C in a desiccator. Esterase activity was evaluated every 10 days. All results are presented as the mean of three assays.

### 2.7. Preparation of the Racemic Alcohol

The synthesis of (*R,S*)-1-phenylethanol was performed according to Barros-Filho et al., 2010 [32]. The reaction was monitored by TLC analysis until the total disappearance of the acetophenone. The structure of the product was verified by NMR and it was also analyzed with GC, as described in Section 2.10.

### 2.8. Resolution of (*R,S*)-1-Phenylethanol

**Figure 1:**
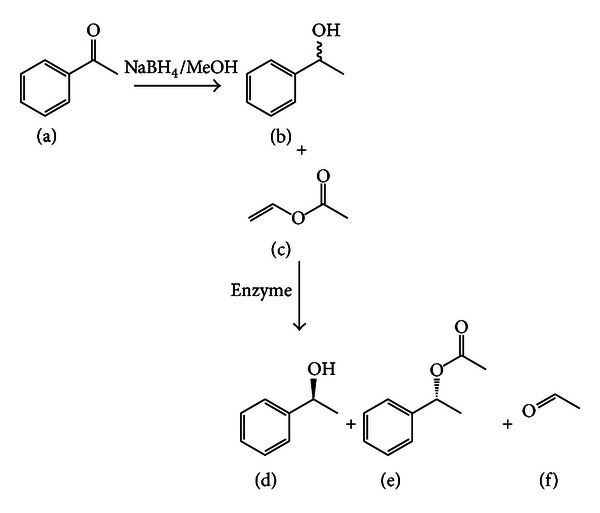
Synthesis of racemic 1-phenylethanol by acetophenone reduction and enantioselective enzymatic resolution by transesterification with vinyl acetate. (a) Acetophenone; (b) (*R,S*)-1-phenylethanol; (c) vinyl acetate; (d) (*S*)-1-phenylethanol; (e) (*R*)-1-phenylethyl acetate; (f) acetaldehyde.

1-Phenylethanol and vinyl acetate as the acyl donor (each 200 mM) were dissolved in 3 mL of hexane. All reactants were previously dehydrated with salt hydrates (saturated solution of LiCl) and molecular sieves (type 4 Å, at a concentration of 10% w/v of the reaction mixture) to get *a*_w_ of 0.15. The reactants were dehydrated until the desired *a*_w_ was reached. Water activity (*a*_w_) was determined using a hygrometer (Awquick, Rotronic Instrument Corp., USA). The reaction was initiated after the addition of enzymes (40 mg of NStcI immobilized or 15 mg of Novozym 435). It was incubated at 37°C, with a shaking speed of 200 rpm on an orbital shaker and the reaction kinetics was followed during 120 h.  Figure 1 shows the steps involved to obtain the product (1-phenylethyl acetate) as a result of the enzymatic resolution. The enantiomeric ratio (*E*) was calculated as proposed by Straathof and Jongejan [33], according to (1) as

(1) $$\begin{array}{c} E=\frac{Ln\left[\left(1-ee_{s}\right)/\left(1+\left({ee}_{s}/{ee}_{p}\right)\right)\right]}{Ln\left[\left(1+ee_{s}\right)/\left(1+\left(ee_{s}/ee_{p}\right)\right)\right]}. \end{array}$$

### 2.9. Effect of Solvent and *a*_w_ on NStcI Enantioselectivity

It was investigated by carrying out the reaction for 120 h in diisopropyl ether and hexane with *a*_w_ of 0.15 and 0.07. The products were evaluated by TLC and GC as described below. All results are presented as the mean of three assays.

### 2.10. Identification of Compounds

All reaction products were analyzed by TLC using a mixture of hexane and ethyl acetate (8 : 2) as solvent; the plates were developed by spraying with ceric sulfate. GC analysis was performed with a chiral column (Chiraldex B-DM (30 m × 0.25 mm × 0.12 *μ*m)) and with a flame ionization detector. The carrier gas was hydrogen. The column temperature was kept at 50°C for 10 min and then it was raised to 130°C at a rate of 7.5°C per min and it was kept at this temperature for 10 min. No reaction was detected in the absence of the enzymes. The structure of the starting alcohol ((*R,S*)-1-phenylethanol) was identified by NMR: the analysis was made in CDCl_3_ at 300 K on a Varian Vxr-300S spectrometer operating at 300 MHz (^1^H). Approximately 10 mg of racemic alcohol were dissolved in CDCl_3_, with tetramethylsilane (TMS) as internal standard. The structure was corroborated by direct comparison of spectral properties. 

## 3. Results and Discussion

### 3.1. NStcI Heterologous Expression

**Figure 2:**
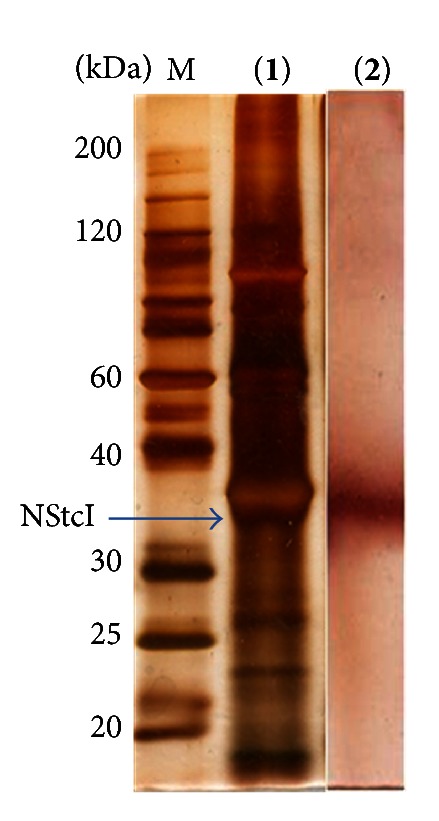
Protein pattern and zymogram of concentrated extracellular extract of recombinant clone expressing NStcI protein in *Pichia pastoris*. (**1**) Protein pattern on SDS-PAGE gel after silver staining. M, low molecular weight marker; NStcI, concentrated crude extract of the recombinant clone expressing NStcI protein. (**2**) Esterase activity on *α*-NA after renaturation of NStcI protein expressed by recombinant clone. The protein band corresponding to NStcI is indicated with an arrow.

*P. pastoris* harboring the *nstcI *gene in its genomic DNA was grown in BMGY medium and then transferred to BMMY medium. Esterase activity towards *p*-NPA (386.4 U/mL) was detected after 48 h of methanol induction and 0.69 mg protein/mL were obtained. Specific activity value (560 U/mg protein) was higher than that in the previously reported results (360 U/mg protein) [18]. This difference may be explained because in this case a different method was used to assay enzyme activity, which uses methanol as solvent; therefore, NStcI activity is improved with this solvent. SDS-PAGE and zymogram analysis showed a single protein band with esterase activity, having a molecular mass of 35 kDa, which corresponds to the theoretically calculated mass of 34.56 kDa and to the experimental molecular mass obtained previously for NStcI (Figure 2) [29]. 

### 3.2. Enzyme Immobilization

An ultrafiltered preparation of the recombinant NStcI enzyme was used for the immobilization procedure, which was carried out by adsorption. Hydrophobic interactions are the dominating adsorption forces between hydrophobic supports and proteins. Initially, the immobilization process of NStcI in polypropylene Accurel MP1000 resulted in a decrease of esterase activity after adsorption giving an immobilization efficiency of 62.57% (Table 1). The immobilization process was optimized by varying protein/support ratio and adsorption time conditions. As a result, immobilization of NStcI was successfully performed with an immobilization efficiency of 81.94% (based on esterase activity), and an amount of adsorbed enzyme of 4 mg of adsorbed protein/g of support (Tables 1 and 2). Lipases are selectively adsorbed onto Accurel MP1000, a feature that results in the purification of the lipase as well as in its immobilization, due to their hydrophobic domains [6–9, 34]. May be we have obtained this behavior after adsorption of NStcI esterase as we have a low protein yield (42.48%), while immobilization efficiency was high (81.94%) (Table 1). Some authors have reported hyperactivation (approximately 240%) of esterase after immobilization by adsorption [9], but in this case it was not detected. However, almost all initial esterase activity was retaining after immobilization (Table 2). A similar behavior has been usually observed for other immobilized CEH, like the *Lactobacillus plantarum *esterase and lipase from *Bacillus thermocatenulatus* [6, 35]. 

### 3.3. Operational and Storage Stability of Immobilized Carboxylesterase

For any subsequent industrial application, the stability of the immobilized enzyme is extremely important [36]. The immobilized esterase NStcI was stable after 3 cycles of *p-*NPA hydrolysis retaining more than 70% residual activity, which is according to the results obtained for other immobilized enzymes through adsorption methods. This establishes the effectiveness of immobilization and the reusability of the NStcI esterase [37, 38]. The reason for the decay of activity was explored and it was not due to enzyme desorption from Accurel MP1000 because no increase in protein concentration in supernatant was detected after each washing of the immobilized esterase. As a consequence, this could be due to an intrinsic loss of activity. 

The immobilized enzyme on Accurel MP1000 retains 100% of its initial activity for 40 days of storage at both at 4°C and RT. These results indicate a great stability of the biocatalyst and are in agreement with Branco and coworkers, who found that immobilized enzymes on a hydrophobic support have greater storage stability. An explanation for this behavior is that, while, there is more hydrophobic support, it will retain less water, and therefore all the deactivation processes related to the degree of hydration will be less likely, ensuring a better preservation of the enzyme structure [39].

### 3.4. Biochemical Characterization

#### 3.4.1. Effects of Temperature and pH on Immobilized Enzyme Activity and Stability

**Figure 3:**
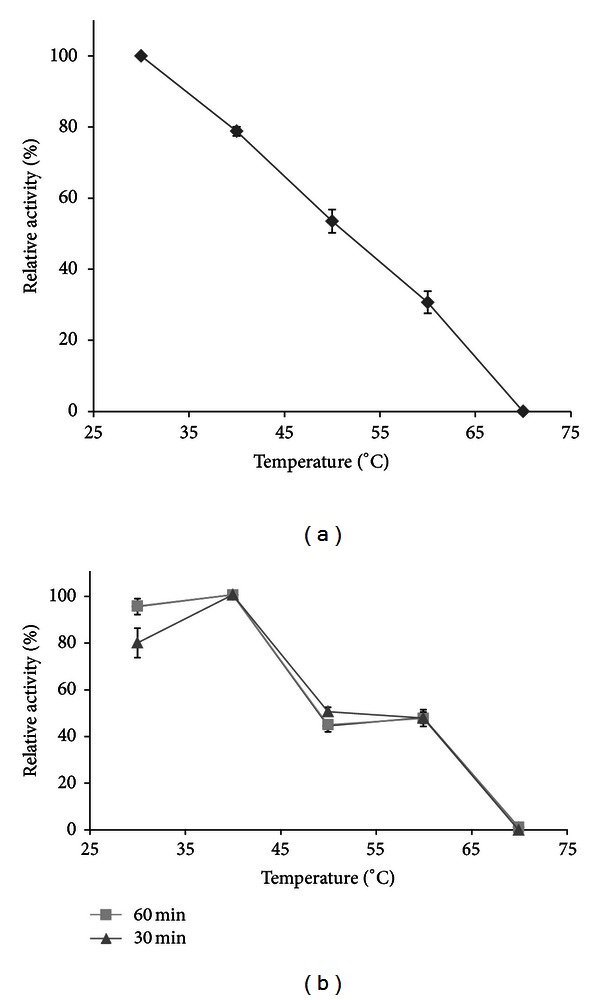
Optimum (a) and stability (b) temperature for the immobilized NStcI esterase.

The analysis of Figure 3 shows that the optimal temperature for the immobilized enzyme on Accurel MP1000 was 30°C and the thermal stability was in the range of 30–40°C after 1 hour. However, it is important to note that, after incubation at 50 and 60°C for 1 h, the enzyme can retain 47.5% of its initial activity. Previous results for free recombinant NStcI enzyme displayed that it was more active in temperature ranges of 30–50°C, showing a maximum activity at 40°C. The thermostability of NStcI after 30 min incubation showed that it can retain up to 70% of their initial activity in the range of 30–40°C. However, when the temperature was elevated to 50°C, free NStcI lost 50% of its initial activity and at 60°C no activity was observed [29]. The results displayed in this work are in agreement with those previously obtained, with the exception that thermostability of NStcI increased after immobilization on Accurel MP1000. 

**Figure 4:**
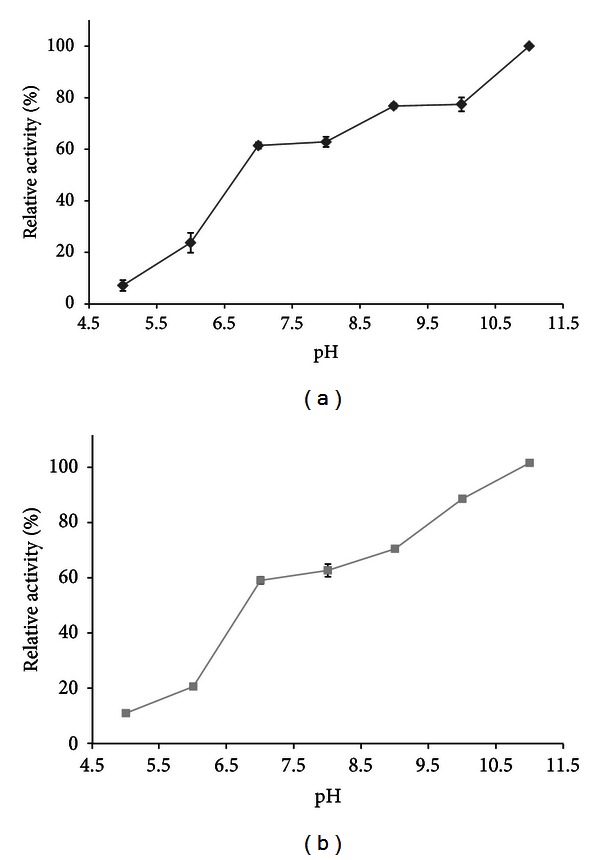
Optimum (a) and stability (b) pH for the immobilized NStcI esterase.

Immobilized NStcI was active in the pH range of 7–11 (Figure 4), with a maximum at pH of 11. After a 12 h incubation period at different pH values, NStcI was more stable in the pH range of 7–11 and the highest retained activity was also at pH of 11. In contrast, results previously obtained for the free recombinant NStcI esterase showed that it was active in the pH range of 7–9 and it was more stable in the pH range of 7–10 after 12 h incubation [29]. Consequently, immobilized NStcI increased its stability at alkaline pH.

#### 3.4.2. Different Chain-Length Specificity for Immobilized NStcI Esterase

**Figure 5:**
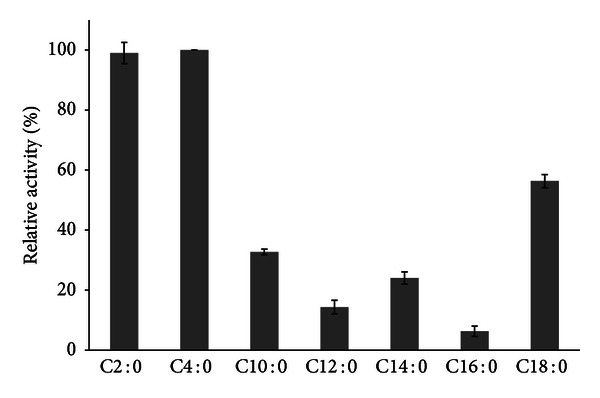
Substrate specificities of the immobilized NStcI esterase against *p*-NP esters of different chain length.

The immobilized NStcI esterase was able to hydrolyze all *p*-nitrophenyl synthetic substrates evaluated (Figure 5). The highest activity was observed towards C2:0 chain-length *p*-nitrophenyl ester (*p*-NPA) which is in accordance with the previously reported results. However, immobilized NStcI esterase was able to hydrolyze also C4:0 chain-length *p*-nitrophenyl ester (*p*-NPB) so efficiently as for *p*-NPA, which was not observed for the free enzyme. Another observed difference was that the hydrolysis of middle chain-length esters was also increased when compared to the results reported for the free enzyme [29].

#### 3.4.3. Influence of Solvents on Hydrolysis Activity

**Figure 6:**
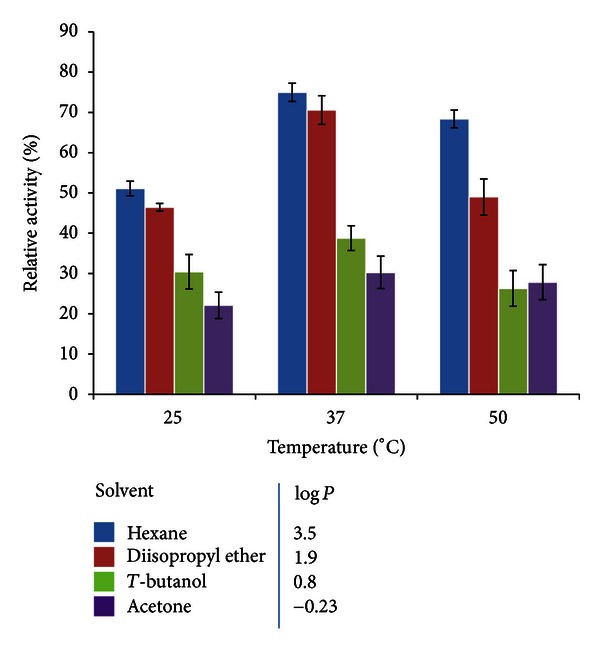
Effect of temperature and solvents on stability of the immobilized NStcI esterase.

Aqueous solutions are the natural environment for enzyme reactions. Nevertheless, it is often useful to change to nonaqueous media when employing enzymes in organic chemistry [24]. The enzymatic stability in organic media can vary with the solvent used, thus the correct selection can only be made after the evaluation of different solvents. The stability of immobilized NStcI esterase was examined under different combinations of temperature and solvents. After 72 h of incubation, its residual activity was assayed with *p-*NPA as substrate, as shown in Figure 6. A correlation between the log *P* of the solvent and esterase stability was observed. Hydrophobic solvents (hexane and diisopropyl ether) favored higher enzyme activity than the hydrophilic ones (*t*-butanol and acetone). Our results are in accordance with those observed for many enzymes in organic solvents. Hydrophilic solvents, like acetone, can take up infinite amounts of water and they strip the remaining water molecules off the enzyme surface [40]. Similar results have been reported for some commercial lipases [41]. Hydrophilic solvents can also deactivate enzymes by disrupting the functional structure, an irreversible process. This is the case of hen egg-white lysozyme; after evaluation of its solubility in a wide range of solvents, it has been demonstrated that organic solvents have little effect on suspended lyophilized enzyme, whereas hydrophilic solvents tend to dissolve the protein and severely disrupt its tertiary structure [42]. For proteases, a study of long-term stability in organic media showed that after returning of the enzymes to an aqueous medium, the catalyst lost most of its aqueous activity. These authors conclude that the process leading to inactivation of proteases in organic media is clearly irreversible even on return to water, contrary to the expected reversion of inactivation when it is simply due to dehydration by continuous operation in hydrophilic solvents [43]. 

Interestingly, the opposite effect was observed for the free enzyme after 72 h, where hydrophilic solvents such as *t*-butanol and acetone resulted in higher enzyme activity (data not shown). This behavior has been scarcely observed for some lipases, such as those from *Serratia marcescens *and *Bacillus sp*. 42, as well as for an esterase of *Arthrobacter nitroguajacolicus* [44–46]. In the case of a porcine pancreatic lipase, it has been also demonstrated that the water is so tightly bound to the enzyme that even hydrophilic solvents do not strip it. Hence, the main factor in the effect of organic solvents seems to be not their interaction with the enzyme molecule itself but their interaction with the enzyme-bound water [47]. 

The immobilized NStcI showed the highest stability and activity at 37°C, probably due to the fact that a better protein conformation and flexibility is achieved, and these results are consistent with those previously reported for the free enzyme [29]. The solvent which has the highest activity reached was hexane. We evaluated the temperature that increased to 50°C and NStcI retains 68% of its activity in hexane. Interestingly, it is important to mention that again immobilized enzyme showed better temperature stability than the free enzyme. Previously reported results showed that at 50°C, the enzyme could retain only 43% of activity obtained at 37°C, which is the temperature at which the highest activity is observed [29]. Therefore, synthesis reactions were performed at 37°C in hexane, where the enzyme retained 74% of its initial activity after 72 h. 

### 3.5. Chemical Synthesis and Characterization of Substrate ((*R,S*)-1-Phenylethanol)

Acetophenone was reduced and the presence of the alcohol was determined by means of TLC. The identity of the (*R,S*)-1-phenylethanol was confirmed by ^1^H NMR spectroscopy. The signals were in agreement with data already reported [48]. According to GC analysis, the retention time is for (*S*)-1-phenylethanol and (*R*)-1-phenylethanol were 19.2 and 18.9 min, respectively. 

### 3.6. Influence of Time of Reaction on Resolution of (*R,S*)-1-Phenylethanol

**Figure 7:**
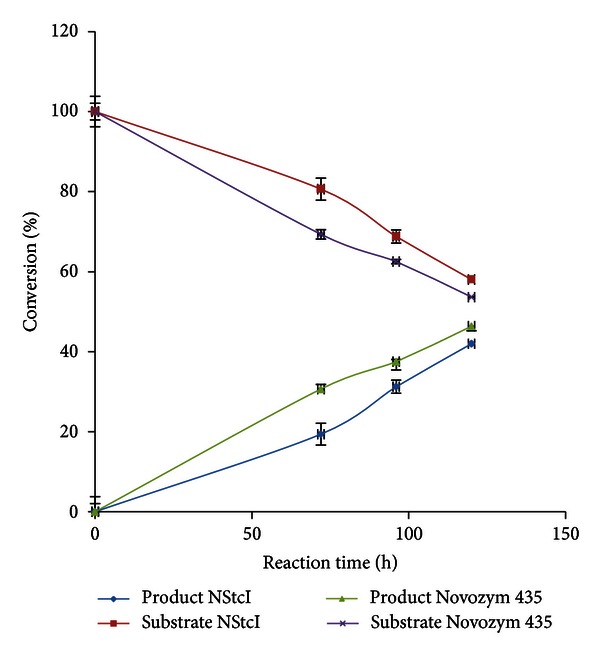
Substrate consumption by NStcI (■) or Novozym 435 (x) and product generation by NStcI (◆) or Novozym 435 (▲) during resolution of (*R,S*)-1-phenylethanol by the immobilized NStcI esterase at different times of reaction.

According to the results obtained for enzyme stability in Section 3.4, only hydrophobic solvents were chosen for resolution assays. Initially, hexane was evaluated as reaction medium and the results showed that both NStcI esterase from *A. nidulans *and a positive control (commercial lipase Novozym 435) catalyzed the transesterification of racemic 1-phenylethanol with vinyl acetate to (*R*)-enantiomer. The retention times of (*R*)-1-phenylethyl acetate, (*R*)-1-phenylethanol, and (*S*)-1-phenylethanol were 18.7, 18.9, and 19.2 min, respectively. The enantiomeric excess (*ee*_*p*_) is dependent on the reaction time. In Table 3, the time course of transesterification revealed that the maximum conversion degree was obtained for both enzymes at 120 h of reaction and they were close to the theoretical value (50%) (Figure 7) [14]. 

### 3.7. Influence of Water Activity on the Enantioselectivity

Water activity plays an important role in enzyme-catalyzed reactions. The influence of thermodynamic water activity on the enantioselectivity of enzymes was investigated in this study. The calculated enantiomeric ratio (*E*) indicates that by decreasing *a*_w_ from 0.15 to 0.07, the enantioselectivity for NStcI increased 2.54-fold, while for Novozym 435 it increased 3.51-fold (Table 3). At *a*_w_ of 0.07 and after 120 h of bioconversion in hexane, the highest possible conversion under assayed conditions was reached by both biocatalysts (42% for NStcI and 46% for Novozym 435) with enantiomeric excess for reactants of 71.7% for NStcI and 85.5% for Novozym 435. These results are in accordance with the previously obtained by Léonard et al. [49]. They studied the effect of *a*_w_ on *Candida antarctica* Lipase B (CALB) enantioselectivity for the acylation of pentan-2-ol with methylpropanoate and their results show that in the *a*_w_ interval 0 to 0.2, the enantioselectivity increased from 101 to 320, but at higher *a*_w_ values enantioselectivity decreased. They also found, by means of molecular modeling, that bound water molecules in the stereospecificity pocket of CALB would inhibit (*S*)-enantiomer reaction, while it would not influence (*R*)-enantiomer. Therefore, water may act as a competitive and enantioselectivity inhibitor for the resolution of (*R,S*)-1-phenylethanol with NStcI and Novozym 435. There are reports of optimization of kinetic resolution of (*R,S*)-1-phenylethanol over carboxylesterases in ionic liquids [12, 50]. The optimization of enzymatic kinetic resolution of (*R,S*)-1-phenylethanol over *Candida antarctica* lipase B (CALB) (Novozym 435) renders the highest possible conversion (50%) with enantiomeric excess for substrate higher than 99% [12]. On the other hand, *Bacillus pumilus* lipase is enantioselective for the hydrolysis of (*R,S*)-1-phenylethyl acetate (*ee* = 89%) [50]. The results obtained in the present work (conversion of 42% for NStcI and 46% for Novozym 435, with enantiomeric excess for reactants of 71.7% for NStcI and 85.5% for Novozym 435) are close to those obtained for the lipases of *C. antarctica* and *B. pumilus* in ionic liquids. However, it is necessary to do a further optimization of reaction parameters, such as enzyme concentration, ionic liquid as reaction medium, and substrates concentrations with respect to the final product concentration, which could yield even better results. In a recent published work, it was reported *E* values in excess of 200 for lipases [51].

### 3.8. Effect of Organic Solvent on Enantioselective Synthesis

It is known that the solvent can affect the enantioselectivity in an enzymatic resolution [25, 26]. Even though another hydrophobic solvent was assayed as reaction medium (diisopropyl ether), the results in Table 3 showed that the enantioselectivities of the both enzymes decreased significantly. For the reaction catalyzed by Novozym 435, *E* value decreased 3.1-fold, while for NStcI was zero. Molecular modeling studies of Graber et al. [52] suggest that molecules of solvent can remain in the catalytic site of enzyme and form hydrogen bonds with the oxyanion hole and/or the catalytic serine; in their research polar solvents showed more affinity for the active site than nonpolar solvents. According to these results, molecules of diisopropyl ether could be binding to the catalytic site of enzymes because the solvent is more hydrophilic than hexane. Diisopropyl ether could perturb the protein conformation and flexibility, in the way of stripping off the essential water layer of the enzyme. As solvent has a more hydrophilic tendency to strip some of this water, consequently, the catalytic activity decreases due to the lack of bound water to maintain the enzyme flexibility [40].

## 4. Conclusions

In this work, a new catalyst is described, the recombinant NStcI immobilized in Accurel MP1000. Temperature and pH stability of NStcI esterase have been improved trough the immobilization process. Only few microbial carboxylesterases have been used in synthesis reactions and they show moderate enantioselectivity, and then new enzymes like NStcI are needed. In addition to the chemio- and regio-selectivity already reported for NStcI, we have demonstrated its enantioselectivity for an important industrial synthesis reaction. This property is strongly related to the nature of solvent and the medium *a*_w_. The obtained results for this new recombinant enzyme are useful even though this has not been optimized yet for a large scale process. It would be important to evaluate, with this new biocatalyst, the resolution of other interesting racemic mixtures.

## Figures and Tables

**Table 1:** Immobilization efficiency (*E*%) and protein yield (*P*%) of immobilized recombinant esterase NStcI from *A. nidulans* on Accurel MP1000.

Immobilization conditions	^†^ *P* (%)	*E (%)
A	94.11 ± 2.26	62.57 ± 1.37
B	42.48 ± 1.52	81.94 ± 2.65

The esterase and protein assays were performed as described in Section 2.

*E%. It was calculated as described in the following equation: *E* (%) = ((*U*_*E*_ − *U*_*I*_)/*U*_*I*_) · 100, where *U*_*E*_ is the total units of activity added for immobilization procedure; *U*_*I*_ is the total units of activity in the solution after immobilization procedure.

^†^ *P*%. It was calculated as described in the following equation: *P* (%) = ((*P*_*E*_ − *P*_*I*_)/*P*_*I*_) · 100, where *P*_*E*_ is the total protein added for immobilization procedure; *P*_*I*_ is the total protein in the solution after immobilization procedure.

**Table 2:** Yields of the immobilization of NStcI esterase.

	Total protein (mg)	Total activity (U)	Specific activity (U/mg of protein)
Free esterase	9.42 ± 0.38	4335.80 ± 14.22	459.40 ± 0.18
Supernatant	5.42 ± 0.27	783.07 ± 1.12	130.43 ± 0.32
Immobilized esterase	4.00 ± 0.15	3552.80 ± 21.45*	412.50 ± 0.63^†^

*This value was obtained by difference between the free esterase solution and supernatant.

^†^Activity value was obtained experimentally as described in Section 2.4.

**Table 3:** Effect of solvent and *a*_w_ on the enzymatic resolution of (*R*/*S*)-1-phenylethanol by esterase NStcI.

Solvent	*a*_w_ initial	Reaction time (h)	Enzyme	%*ee*_*p*_	*E*	%*C*_*p*_
Hexane	0.15	96	NStcI	45.1 ± 5.8	12.3 ± 2.1	31.2 ± 2.7
			Novozym 435	59.4 ± 2.9	19.2 ± 1.8	37.5 ± 1.1
	0.07	120	NStcI	71.7 ± 4.9	31.3 ± 6.9	42.0 ± 1.6
			Novozym 435	85.5 ± 1.9	67.5 ± 9.9	46.3 ± 0.5

Diisopropyl ether	0.07	120	NStcI	0	0	0
			Novozym 435	62.4 ± 2.0	21.3 ± 1.5	38.7 ± 0.7

All results were represented as the mean ± S.D. of 3 separated determinations.

## Abbreviations

Eq: Equation
*α*-NA: *α*-Naphthyl acetate
*p*-NPE: *p*-Nitrophenyl esters
*p*-NPA: *p*-Nitrophenyl acetate
*p*-NP: *p*-Nitrophenol
RT: Room temperature
NMWL: Nominal molecular weight limit
SDS-PAGE: Sodium dodecyl sulphate polyacrylamide gel electrophoresis
TLC: Thin layer chromatography
NMR: Nuclear magnetic resonance
GC: Gas chromatography.
